# Identification of a SARS-CoV-2 virus-derived vmiRNA in COVID-19 patients holding potential as a diagnostic biomarker

**DOI:** 10.3389/fcimb.2023.1190870

**Published:** 2023-06-02

**Authors:** Qian Zhao, Jinhui Lü, Bing Zhao, Yuefan Guo, Qiong Wang, Shanshan Yu, Lipeng Hao, Xiaoping Zhu, Zuoren Yu

**Affiliations:** ^1^ Research Center for Translational Medicine, Shanghai East Hospital, Tongji University School of Medicine, Shanghai, China; ^2^ Microbiological Testing Lab, Shanghai Pudong Center for Disease Control & Prevention, Shanghai, China; ^3^ Department of Respiration, Shanghai East Hospital, Tongji University School of Medicine, Shanghai, China

**Keywords:** SARS-CoV-2, vmiRNA, COVID-19, biomarker, small RNA

## Abstract

Severe acute respiratory syndrome coronavirus 2 (SARS-CoV-2) has become a lasting threat to public health. To minimize the viral spread, it is essential to develop more reliable approaches for early diagnosis of the infection and immediate suppression of the viral replication. Herein, through computational prediction of SARS-CoV-2 genome and screening analysis of specimens from covid-19 patients, we predicted 15 precursors for SARS-CoV-2-encoded miRNAs (CvmiRNAs) containing 20 mature CvmiRNAs, in which CvmiR-2 was successfully detected by quantitative analysis in both serum and nasal swab samples of patients. CvmiR-2 showed high specificity in distinguishing covid-19 patients from normal controls, and high conservation between SARS-CoV-2 and its mutants. A positive correlation was observed between the CvmiR-2 expression level and the severity of patients. The biogenesis and expression of CvmiR-2 were validated in the pre-CvmiR-2-transfected A549 cells, showing a dose-dependent pattern. The sequence of CvmiR-2 was validated by sequencing analysis of human cells infected by either SARS-CoV-2 or pre-CvmiR-2. Target gene prediction analysis suggested CvmiR-2 may be involved in the regulation of the immune response, muscle pain and/or neurological disorders in covid-19 patients. In conclusion, the current study identified a novel v-miRNA encoded by SARS-CoV-2 upon infection of human cells, which holds the potential to serve as a diagnostic biomarker or a therapeutic target in clinic.

## Introduction

Since the outbreak of coronavirus disease 2019 (covid-19) in early 2020, severe acute respiratory syndrome coronavirus 2 (SARS-CoV-2) has kept the epidemic going globally for three years through its cunning method of mutation. Up to the end of 2022, there have been 660 million confirmed cases, including 6.7 million deaths, across ~200 countries. The global economy suffered too much from the pandemic. It is essential to determine the pathological mechanisms and develop new conservative biomarkers for early diagnosis and immediate treatment after infection.

MicroRNAs (miRNAs), a class of small non-coding RNAs with approximately 18−25 nucleotides in length, are believed to regulate diverse biological processes and various human diseases by regulating gene expression through translational inhibition and/or epigenetic modification. Viruses cleverly use miRNA regulatory systems as a strategy to circumvent host defense mechanisms and regulate fundamental biological processes. Since the first report of virus-derived miRNAs (v-miRs) in the Epstein-Barr virus (EBV) infected cells (Pfeffer et al., 2004), evidence have demonstrated that viruses could encode v-miRs using either canonical or non-canonical miRNA biogenesis pathways (Tycowski et al., 2015). A number of RNA sequencing data have revealed a new non-canonical approach for v-miR biogenesis independent of nuclear microprocessors (Ruby et al., 2007; Babiarz et al., 2008). Cytoplasmic translocation of Drosha during viral infection may be a mechanism for the generation of v-miRs derived from RNA viruses (Shapiro et al., 2012). So far, around 1,300 mature v-miRs have been registered in the VIRmiRNA database (Qi et al., 2006; Qureshi et al., 2014). Although v-miRs derived from DNA viruses have been well described, RNA virus-encoded v-miR study remains at the beginning stage.

A few RNA viruses have been reported to encode v-miRs including West Nile virus (WNV) (Hussain et al., 2012), HIV retrovirus (Omoto and Fujii, 2005; Kaul et al., 2009), Ebola filovirus (Liang et al., 2014; Chen et al., 2016; Duy et al., 2018), and H5N1 influenza virus (Li et al., 2018). v-miRs may circumvent the immune response and extend the longevity of infected cells. For example, KUN-miR-1 encoded by WNV Kunjin was reported to reduce host resistance and promote viral infection, as was HIV1-miR-H1 encoded by HIV (Brennecke et al., 2005; Kaul et al., 2009; Hussain et al., 2012). In addition, circulating v-miRs have been successfully detected in serums of the infected patients, such as circulating vmiRNA-367 encoded by HIV-1 (Omoto et al., 2004) and circulating miR-VP-3p encoded by the Ebola virus (Chen et al., 2016).

v-miRs encoded by SARS-CoV-2 have been reported recently by literature (Arisan et al., 2020; Sacar Demirci and Adan, 2020) and our previous study (Zhao et al., 2022a). MR147-3p was primarily predicted from SARS-CoV-2 viral genome and further validated in the SARS-CoV-2-infected Vero E6 cells (Liu et al., 2021). Zheng et al. identified SARS-CoV-2-encoded miR-nsp3-3p in the serum of patients (Fu et al., 2021). Our previous work identified CvmiR-5-5p in the patients’ serums and sputum samples (Zhao et al., 2022a). The current study identified a novel v-miR, named CvmiR-2, in the nasal swab and serum samples of covid-19 patients, which was specifically encoded by SARS-CoV-2, and highly conserved between SARS-CoV-2 and its mutants. It showed higher levels in severe patients, compared to non-severe ones. Transfection of the CvmiR-2 precursor into human alveolar basal epithelial cell line A549 led to biogenesis of mature CvmiR-2. 1,248 human genes were predicted as targets of CvmiR-2. Pathway analysis suggested CvmiR-2 involvement in the regulation of the immune response, muscle pain, and/or neurological disorders in patients. The current study not only identified a novel v-miR encoded by SARS-CoV-2, but also provided a potential target for early diagnosis and/or gene therapeutic of covid-19 patients.

## Materials and methods

### Sample collection

Patient samples were collected by Shanghai Pudong Center for Disease Control & Prevention. Negative control samples were collected by Shanghai East Hospital. All the procedures were approved by the Institutional Review Board (IRB) of Shanghai East Hospital (2022-Yanshen-013). All patients were provided with a written informed consent form.

### CvmiRNAs prediction

The genome sequence of the SARS-CoV-2 virus (MN908947.3) was used to predict vmiRNAs by using miRPara6.3 (Wu et al., 2011). SVM probability >0.99 was applied for parameter filtering of mature vmiRNAs (Zhao et al., 2022a). The website of mfold (http://www.unafold.org/) was employed to predict the secondary structure of pre-CvmiRNAs. Pre-CvmiR sequence we identified has been accepted in GenBank (GenBank ON124949 - ON124951). Pre-CvmiR-2 is available at https://www.ncbi.nlm.nih.gov/nuccore/ON124949.

### Cells, vectors, and transfection

A549 cells were originally purchased from ATCC, and cultured in DMEM containing 10% FBS (Gibco, USA), penicillin (100 U/mL), and streptomycin (100 μg/mL). The sequence of pre-CvmiR-2: 5’ AUCAAACGUUCGGAUGCUCGAACUGCACCUCAUGGUCAUGUUAUGGUUGAGCUGGUAGCAGAACUCGAAGGCAUUCAGUACG 3’, which was synthesized by GeneScript (Nanjing, China), and cloned into pcDNA3.1 plasmid. An empty vector was used as a negative control. pcDNA3.1-pre-CvmiR-2/NC plasmid was transfected into A549 cells for 24 hours according to the manufacturer’s instructions of Lipofectamine 2000 (Invitrogen) with a final concentration of 0.25 μg/mL.

### QRT-PCR analysis

Total RNA of patient samples was extracted using MagNA Pure 96 DNA and Viral NA Small Volume Kit (Roche, Mannheim, Germany) and MagNA Pure 96 Instrument (Roche, Mannheim, Germany) following the manufacturer’s instruction. Total RNA of A549 cells was extracted using Trizol reagent (Invitrogen, United States) according to standard procedure. Covid-19 Real-Time PCR Kit (BioGerm, Shanghai, China) was used for lab testing. CvmiRs were quantitatively analyzed as described in our previous publication (Zhao et al., 2022b). 50-200 ng of total RNA was first treated with DNase I (Promega, USA) and further used to prepare the first-strand cDNA of CvmiRs using the M&G miRNA Reverse Transcription Kit (miRGenes, China) according to the manufacturer’s instructions. The SYBR Green Master Mix (Applied Biosystem, United States) and QuantStudio™ 6 Flex Real-Time PCR System (Applied Biosystem, United States) were used for real-time PCR analysis. Then, 5s rRNA and hsa-miR-16 were used as internal controls for normalization. Forward primer sequences for CvmiR-2: 5′ GGUAGCAGAACUCGAAGGCA 3′; Ctrl1: 5′ TGGTCATGTTATGGTTG 3′; Ctrl2: 5′ ATCAAACGTTCGGAT 3′; 5s rRNA, 5′ AGTACTTGGATGGGAGACCG 3′; hsa-miR-16: 5′ TAGCAGCACGTAAATATTGGCG 3′. All primers were synthesized by GenScript (Nanjing, China).

### MiRNA target prediction and pathway analysis

The software of miRanda was applied to predict human target genes of CvmiR-2. The online tool of WebGestalt (http://www.webgestalt.org/) was used to perform the pathway analysis. An online tool (http://www.bioinformatics.com.cn) was applied to generate the pathway color scale bar graph.

### Public dataset

Datasets (GSE148729, GSE183280) of small RNA sequencing of SARS-CoV-2 virus-infected Calu-3 cells (Wyler et al., 2021) or A549-hACE2. Calu3 and PC-9 were used for CvmiR validation. An online tool (http://www.bioinformatics.com.cn) was applied to generate the sequence logo diagrams. Datasets (GSE176498, GSE166160) of small RNA sequencing of COVID-19 patients’ samples were applied to analyze the correlation between CvmiR-2 expression and severity of patients. An RNA-seq dataset of SARS-CoV-2-infected iPS-derived AT2s (iAT2s) was used to derive differentially expressed mRNAs (Hekman et al., 2020).

### Statistical analysis

Data are presented as mean ± SEM unless otherwise stated. Standard two-tailed student’s t-test was applied for statistical analysis, in which p<0.05 was considered as significant.

## Results

### Prediction of SARS-CoV-2-encoded v-miRs

**Figure 1:**
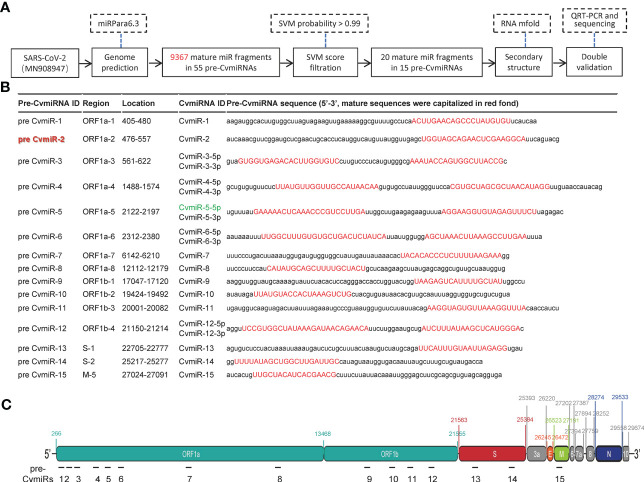
Prediction of SARS-CoV-2-encoded v-miRs. **(A)** The flow chart for the v-miR analysis. **(B)** The sequence information of the 15 predicted v-miR precursors. 20 predicted mature v-miRs were highlighted in red. **(C)** Schematic indication of the 15 v-miR precursor locations in the genome of SARS-CoV-2.

A total of 9,367 mature miRNA sequences in 55 precursor fragments were predicted by screening analysis of the SARS-CoV-2 viral genome using the miRPara6.3 tool as shown in **Figure 1A** and **Supplemental Table S1**. After a cut-off with an SVM probability score>0.99, twenty mature miRNA sequences (named CvmiRNAs) from the 15 precursors were considered as SARS-CoV-2-encoded v-miR candidates (**Figure 1B**). The location and sequence information of the 15 v-miR precursors were indicated in **Figures 1B, C**. The hairpin secondary structure of the 15 precursors was formed using the mFold program, and shown in **Supplemental Figure S1**, in which the mature sequences of CvmiRNAs at either 5’ and/or 3’ arms were highlighted. CvmiR-2 was selected for further analysis in the current study due to its sequence conservation in SARS-CoV-2 and mutants.

### Sequence conservation of CvmiR-2 in SARS-CoV-2 and its mutants

**Figure 2:**
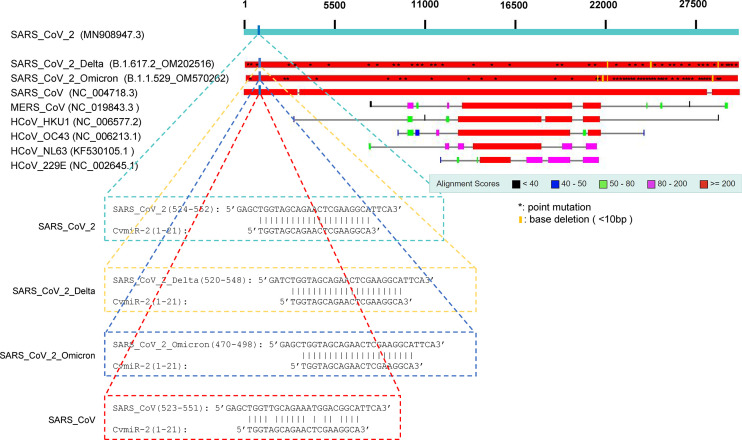
Conservation of CvmiR-2 in SARS-CoV-2 and its mutants. Sequence alignment analysis between CvmiR-2 and SARS-CoV-2, SARS-CoV-2_Delta, SARS-CoV-2_Omicron, SARS_CoV, MERS_CoV, HCoV_HKU1, HCoV_OC43, HCoV_NL63, and HCoV_229E.

By applying a sequence blast analysis of 9 kinds of known coronaviruses, we found that the sequence of CvmiR-2 was specific to the SARS-CoV-2 family, highly conserved (100% conservation) between SARS-CoV-2 and its two known mutants including Delta strain and Omicron strain, while poorly conserved (80.9% conservation) between SARS-CoV-2 and SARS-CoV (**Figure 2**). There were no similar sequences detected in the other five coronaviruses, such as MERS_CoV, HCoV_HKU1, HCoV_OC43, HCoV_NL63, and HCoV_229E (**Figure 2**).

### Detection of CvmiR-2 in covid-19 patients

**Figure 3:**
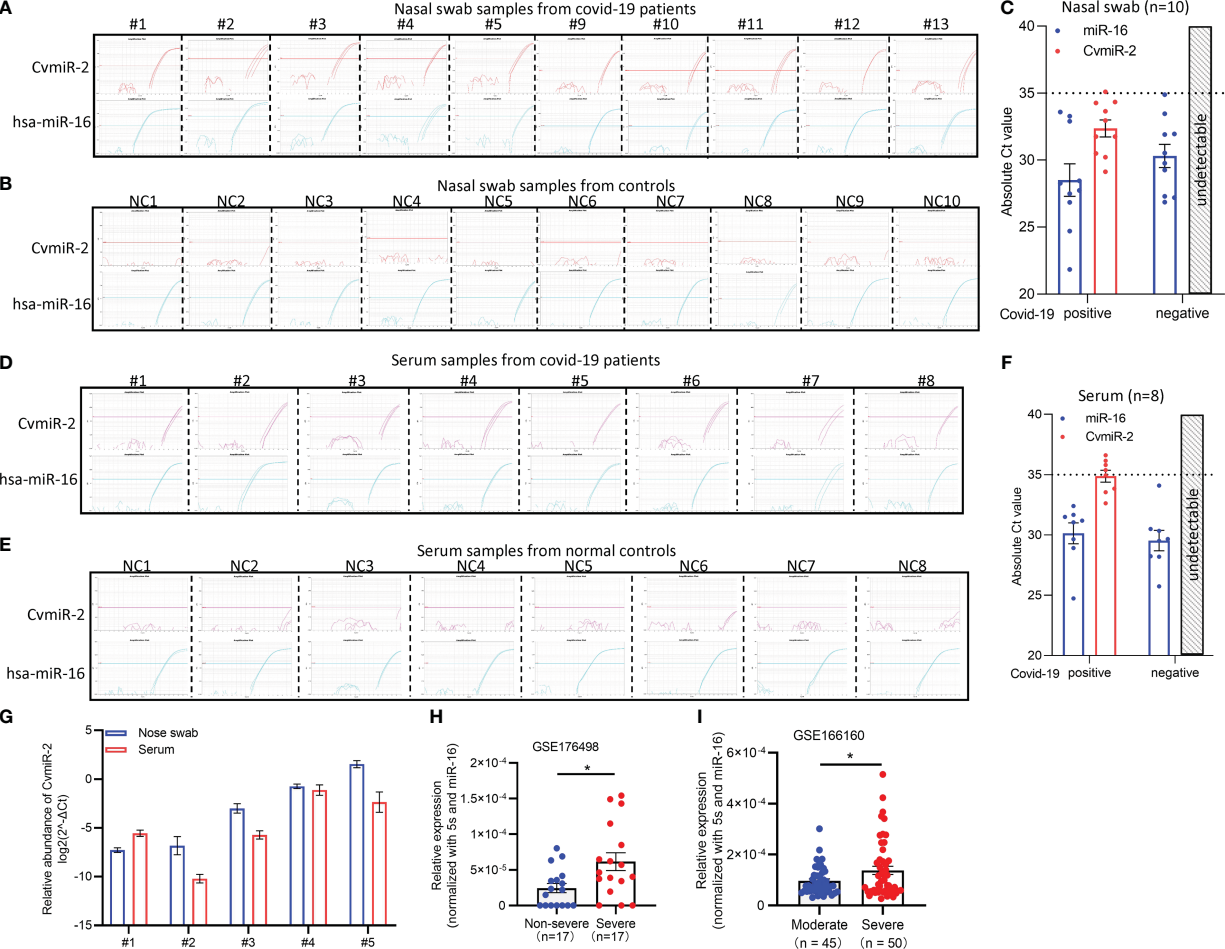
Detection of CvmiR-2 in covid-19 patients. **(A, B)** Amplification curves of CvmiR-2 by QRT-PCR analysis in 10 nasal swab samples from covid-19 patients **(A)** and 10 nasal swab samples from normal controls **(B)**. Hsa-miR-16 served as internal control. **(C)** Quantitative analysis of A and B. Absolute cycle threshold (Ct) values of both CvmiR-2 and hsa-miR-16 were shown. **(D, E)** Amplification curves of CvmiR-2 by QRT-PCR analysis in 8 serum samples from covid-19 patients **(D)** and 8 serum samples from normal controls **(E)**. Circulating hsa-miR-16 served as internal control. **(F)** Quantitative analysis of D and E. **(G)** Comparison of the CvmiR-2 levels in 5 nasal swab samples and matched 5 serum samples from the same patients. **(H)** Higher levels of CvmiR-2 levels in severe patients than that in non-severe patients (n=17 in each group). Data were derived from a public dataset GSE176498. **(I)** Higher levels of CvmiR-2 levels in severe patients (n=50) than that in moderate patients (n=45). Data were derived from a public dataset GSE166160. *p<0.05.

In order to validate the expression of CvmiR-2 in SARS-CoV-2-infected patients, we performed quantitative analysis of CvmiR-2 in 10 nasal swab samples and 8 serum samples from covid-19 patients, and the same number of normal controls as well. Endogenous hsa-miR-16 was used as an internal control. As shown in **Figures 3A–C** and **Supplemental Table S2-1**, CvmiR-2 was amplified in all the 10 nasal swab samples with the absolute Ct (cycle threshold) values between 29 to 35 (**Figures 3A, C**, **Supplemental Table S2-1**), while undetectable in negative controls (**Figures 3B, C**, **Supplemental Table S2-1**). Similarly, we detected the expression of CvmiR-2 in 8 serum samples with absolute Ct values ~32-36 (**Figure 3D**, **Supplemental Table S2-2**), while it was undetectable in all negative controls (**Figures 3E, F**; **Supplemental Table S2-2**). If we set Ct value 35 as a positive signal cutoff, 9 of the 10 nasal swab samples and 4 of the 8 serum samples from patients showed positive, while all the normal control samples showed negative (**Supplemental Table S2**).

We further applied a comparison of CvmiR-2 abundance between the nasal swabs and serum samples. In the 10 nasal swab samples and 8 serum samples above, 5 were matched and collected from the same patients (patient # 1-5). As shown in **Figure 3G**, the CvmiR-2 sensitivity between nasal swabs and serums did not indicate a stable pattern. Analysis of a larger number of samples will be required to come to a conclusion.

In order to identify the relationship between CvmiR-2 expression and the severity of covid-19 patients, a public dataset (GSE176498) of small RNA sequencing profiles in severe and non-severe covid-19 patients was applied. As a result, higher levels of CvmiR-2 were observed in severe covid-19 patients, compared to non-severe ones (**Figure 3H**; **Supplemental Table S3**). An additional dataset (GSE166160) of small RNA sequencing profiles in moderate and severe covid-19 patients further confirmed the positive correlation between the CvmiR-2 expression levels and the disease severity in patients with COVID-19 (**Figure 3I**; **Supplemental Table S4**).

### Validation of CvmiR-2 biogenesis in human cells

**Figure 4:**
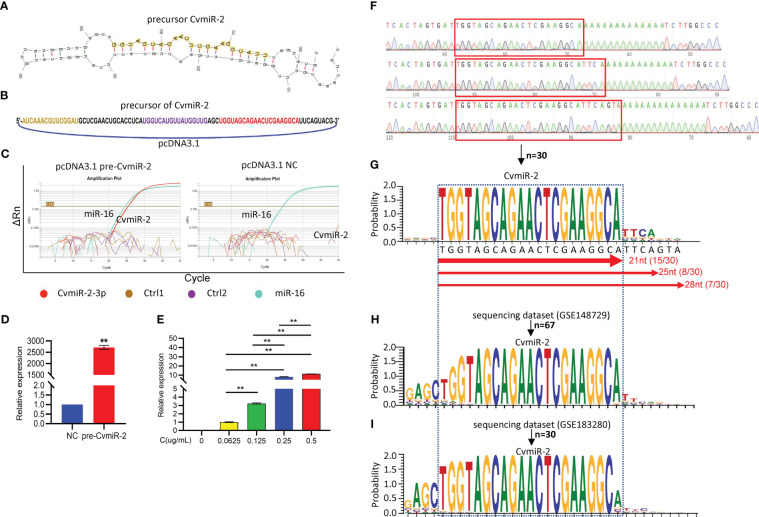
Validation of CvmiR-2 biogenesis in human cells. **(A)** Secondary structure of pre-CvmiR-2. Mature CvmiR-2 was highlighted. **(B)** Structure of the pcDNA 3.1 plasmid carrying pre-CvmiR-2. The sequence of predicted mature CvmiR-2 was highlighted in red. **(C)** QRT-PCR analysis of CvmiR-2 in A549 cells before and after transfection with pre-CvmiR-2. Hsa-miR-16 served as an internal control. Ctr1 and Ctrl2 primers matched to other regions of pre-CvmiR-2. **(D)** The expression of CvmiR-2 in A549 cells with or without transfection with pre-CvmiR-2. **(E)** Quantitative analysis of CvmiR-2 in A549 cells transfected with different concentrations of pre-CvmiR-2. **(F)** Representative sequencing results of CvmiR-2 in C. **(G)** The sequence alignment of CvmiR-2 from 30 clones indicating three isomiRs with 21nt, 25nt, and 28nt in length, respectively. **(H)** The sequence alignment of CvmiR-2 from 67 reads in a public dataset GSE148729 of small RNA sequencing profiles in SARS-CoV-2-infected Calu-3 cells. **(I)** The sequence alignment of CvmiR-2 from 30 reads in a public dataset GSE183280 of small RNA sequencing profiles in SARS-CoV-2-infected PC-9 cells. Data are presented as the mean ± SEM (n=3). **p<0.01.

In order to validate the biogenesis of CvmiR-2 from SARS-CoV-2 upon human infection, the precursor sequence of CvmiR-2 was synthesized and cloned into a pcDNA vector (**Figures 4A, B**), followed by transfection into human alveolar basal epithelial cell line A549. Quantitative PCR analysis demonstrated the biogenesis and exponential amplification of mature CvmiR-2 (**Figures 4C, D**; **Supplemental Table S5**). As expected, its expression exhibited a concentration-dependent pattern (**Figure 4E**).

In order to further validate the sequence of mature CvmiR-2, PCR products of CvmiR-2 in **Figure 4C** were further purified and cloned into TOPO-vector. Thirty clones were randomly selected for sequencing analysis. **Figure 4F** showed representative sequencing results. The sequence alignment demonstrated the enrichment of CvmiR-2 with 21nt in length, and the other two isomiRs with 25nt and 28nt in length, respectively (**Figure 4G**). To double validate the biogenesis and sequence of CvmiR-2 from the SARS-CoV-2 genome, a public dataset (GSE148729) of small RNA deep sequencing profiles in SARS-CoV-2 infected human cells yielded 67 reads, matching both software-predicted and A549-generated CvmiR-2 in sequence (**Figure 4H**; **Supplemental Table S6**). Another dataset (GSE183280) of small RNA sequencing profiles further confirmed the expression of CvmiR-2 in the SARS-CoV-2 infected human cells (**Figure 4I**; **Supplemental Table S7**).

### Correlation between CvmiR-2 and the symptoms

**Figure 5:**
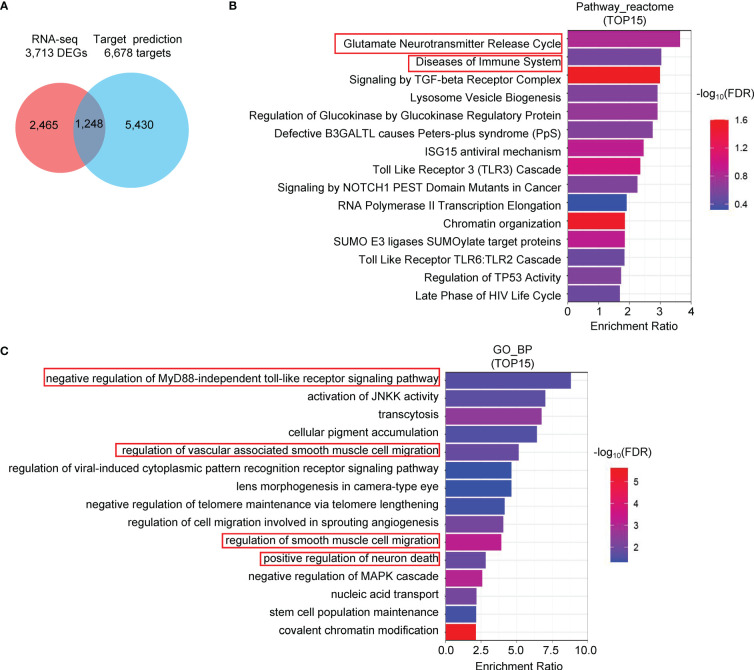
Pathway analysis of the predicted target genes of CvmiR-2. **(A)** 1,248 human genes overlapped between the 6,678 predicted potential target genes of CvmiR-2 and 3,713 DEGs in SARS-CoV-2-infected human cells. **(B, C)** Pathway analyses of the 1,248 genes by Reactome **(B)** and GO **(C)**.

In addition to the diagnostic value, CvmiR-2 may have the potential to be a therapeutic target in the treatment of covid-19 patients. Application of a miRNA target prediction tool (miRanda) predicted 6,678 human genes potentially targeted by CvmiR-2 upon infection. After overlapping with 3,713 DEGs (different expression genes) of RNA-seq data in the SARS-CoV-2-infected iAT2 cells, 1,248 genes were more likely to be the targets of CvmiR-2 in human cells (**Figure 5A**). Reactome pathway analysis of the 1,248 genes suggested regulation of the immune system, toll-like receptors, and neurons (**Figure 5B**, **Supplemental Table S8-1**), which were confirmed by Gene Ontology (GO) analysis (**Figure 5C**, **Supplemental Table S8-2**). In addition, smooth muscle cells showed regulation by CvmiR-2 (**Figure 5C**, **Supplemental Table S8-2**). In view of the common symptoms in covid-19 patients, such as fatigue, muscle pain, neurological disorders, et al, this information may indicate CvmiR-2 involvement in the pathology of the symptoms in covid-19 patients.

Similarly, we applied the target prediction and pathway analysis to the other 19 predicted CvmiRs in **Figure 1B** to indicate their potential in the diagnosis, prognosis and/or treatment of patients with COVID-19 (**Supplemental Table S9**).

## Discussion

Although small non-coding RNAs in mammals have been widely studied with the potential to serve as diagnostic biomarkers or therapeutic targets of diseases, our understanding of the virus-encoded small non-coding RNAs remains very limited, especially for RNA viruses which are not expected to encode small non-coding RNAs to avoid self-degradation. However, emerging evidence has revealed v-miRs derived from multiple types of RNA viruses including Ebola (Chen et al., 2016), H5N1 (Li et al., 2018),and HIV (Schopman et al., 2012).

Only a few studies about small non-coding RNAs encoded by SARS-CoV-2 were reported, in which computational prediction was primarily applied to scan the viral genome, followed by QRT-PCR validation and/or deep sequencing analysis using either serum samples from patients or human cell lines infected by SARS-CoV-2 (Khan et al., 2020; Aydemir et al., 2021; Fu et al., 2021; Li et al., 2021; Liu et al., 2021). MR147-3p and miR-nsp3-3p are the first two small non-coding RNAs identified in SARS-CoV-2-infected cells, holding diagnostic value to predict the risk of covid-19 patients (Fu et al., 2021; Liu et al., 2021). However, the biogenesis of v-miRs from the SARS-CoV-2 virus remains to be determined as yet. The canonical approach for miRNA biogenesis has been well demonstrated, through which pri-miRNAs are further processed to pre-miRNAs to form hairpin structures by the nuclear microprocessor Drosha (Krol et al., 2010; Nanbo et al., 2021). Pre-miRNAs are transported from the nucleolus to the cytoplasm by Exportin-5 and further cleaved by Dicer to form mature miRNAs (Krol et al., 2010). Since the replication of most RNA viruses is completed in the cytoplasm of host cells (Nanbo et al., 2021), the biogenesis of CvmiRs from SARS-CoV-2 may be different from the canonical approach, which is either independent of nuclear microprocessors (Ruby et al., 2007; Babiarz et al., 2008), or relying on the cytoplasmic translocation of Drosha, or through other novel approaches.

Our previous work identified CvmiR-5-5p in covid-19 patients with the potential to mediate the nervous disorder regulation in patients (Zhao et al., 2022a). In order to exclude the amplification of degraded viral fragments, we used a method of adding poly(A) tails to small RNAs, followed by reverse transcription and PCR using an artificial sequence of universal primer. During our CvmiR screening analysis, we tested 20 predicted mature vmiRs by RT-PCR, in which CvmiR-2 and CvmiR-5 showed exponential amplifications with stable signals in covid-19 patients. The current study focused on CvmiR-2 to demonstrate its potential to serve as a diagnostic biomarker or a therapeutic target in COVID-19.

Zheng et al. identified miR-VP-3p in the serum of Ebola virus-infected patients (Chen et al., 2016). A recent report from the same group identified miR-nsp3-3p in the serum of covid-19 patients and demonstrated its potential to distinguish severe patients from mild/moderate ones (Fu et al., 2021). It was thus suggested that virus-derived small non-coding RNAs can serve for earlier diagnosis and risk prediction. In this study, we detected CvmiR-2 expression in both nasal swabs and serum samples of patients. More importantly, it is highly conserved between SARS-CoV-2 and its mutants. A positive correlation between the CvmiR-2 level and patients’ severity was observed, supporting the risk prediction potential of CvmiR-2. Moreover, because of the sequence conservation of CvmiR-2, its biomarker potential may be viral mutants-independent. It is detectable in patients infected by either original SARS-CoV-2 or its variants Delta or Omicron.

We analyzed the potential target genes of CvmiR-2 in human cells. The immune system was identified as one of the main pathways probably regulated by CvmiR-2. In view of the immune response to viral infection, CvmiR-2 may be one of the virus-derived exogenous molecules to activate or suppress the immune response. In addition, regulations of smooth muscle cell migration and neuron death by CvmiR-2 were predicted, suggesting CvmiR-2 involvement in the symptoms in covid-19 patients, such as fatigue, muscle pain, and/or neurological disorders. However, experimental validation will be required before further consideration for translational application.

## Data availability statement

The datasets presented in this study can be found in online repositories. The names of the repository/repositories and accession number(s) can be found in the article/**Supplementary Material**.

## Ethics statement

The studies involving human participants were reviewed and approved by The Institutional Review Board (IRB) of Shanghai East Hospital (2022-Yanshen-013). The patients/participants provided their written informed consent to participate in this study.

## Author contributions

ZY, LH, and XZ designed the research and wrote the paper. QZ, JL, YG, and QW performed gene expression and cellular analyses. BZ and SY collected samples and did part of the analysis; QZ did data analysis. All authors contributed to the article and approved the submitted version.

## Supplementary material

Figure S1 The hairpin secondary structures of the 15 predicted v-miR precursors. The sequences of mature v-miRs were highlighted.

Table S1 List of 9,367 mature miRNA sequences in 55 precursor fragments predicted by screening analysis of the SARS-CoV-2 viral genome using miRPara6.3.

Table S2-1 Ct values in **Figure 3A** and **3B**.

Table S2-2 Ct values in **Figure 3D** and **3E**.

Table S3 Original data of **Figure 3H** (GSE176498).

Table S4 Original data of Figure 3I (GSE166160).

Table S5 Ct values in **Figure 4C**.

Table S6-1 Comparison of predicted CvmiRNA mature sequence and small RNA sequencing results in Calu3 cells infected SARS-CoV-2 for 24 h (GSE148729).

Table S6-2 Original data for Fig 4H (GSE148729).

Table S7-1 Comparison of predicted mature CvmiR sequences and small RNA sequencing results in Calu3, PC-9 and A549-hACE2 cells infected SARS-CoV-2 (GSE183280).

Table S7-2 Original data for Fig 4I (GSE183280).

Table S8-1 Top 15 reactome pathways of 1248 overlapped genes between RNA-seq 3,713 DEGs and 6,678 predicted target genes of CvmiR-2-3p.

Table S8-2 Top 15 GO-BP pathways of 1248 overlapped genes between RNA-seq 3,713 DEGs and 6,678 predicted target genes of CvmiR-2-3p.

Table S9 Target prediction and pathway analysis to other 19 predicted CvmiRs in Figure 1B.
