# Supplementary material for: Identification of a SARS-CoV-2 virus-derived vmiRNA in COVID-19 patients holding potential as a diagnostic biomarker
Source: Front Cell Infect Microbiol. 2023 Jun 2;13:1190870. doi: 10.3389/fcimb.2023.1190870 (PMC10272551; doi:10.3389/fcimb.2023.1190870)

Supplemental Figure S1

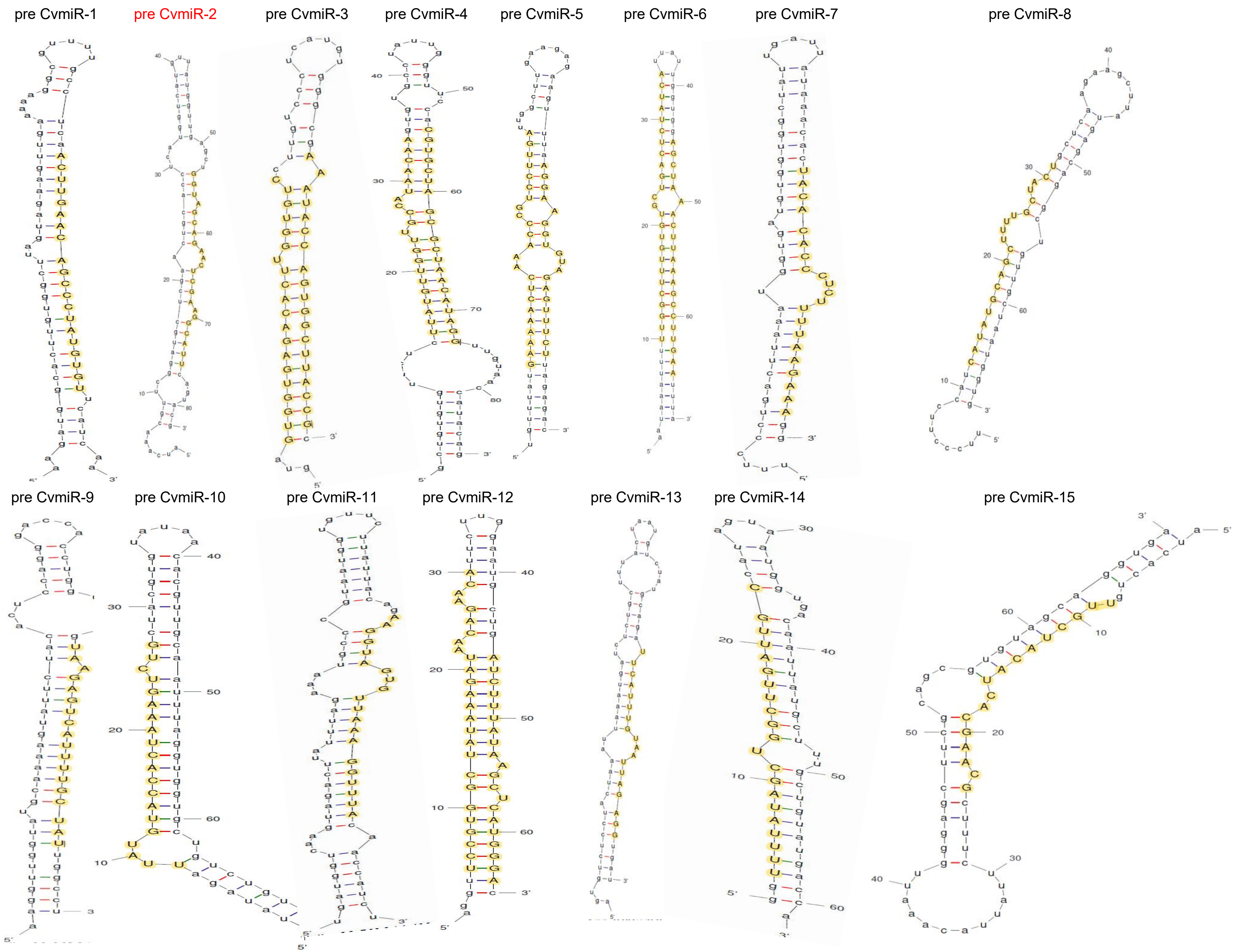

Supplement: Figure S1 — The hairpin secondary structures of the 15 predicted v-miR precursors. The sequences of mature v-miRs were highlighted. [file Image_1.pdf]
